# Admixture mapping of end stage kidney disease genetic susceptibility using estimated mutual information ancestry informative markers

**DOI:** 10.1186/1755-8794-3-47

**Published:** 2010-10-18

**Authors:** Liran I Shlush, Sivan Bercovici, Walter G Wasser, Guennady Yudkovsky, Alan Templeton, Dan Geiger, Karl Skorecki

**Affiliations:** 1 Rappaport Faculty of Medicine and Research Institute, Technion - Israel Institute of Technology and Rambam Medical Center, Haifa 31096, Israel; 2 Faculty of Computer Science, Technion - Israel Institute of Technology, Haifa 32000, Israel; 3 Cabrini Medical Center New York, N.Y., USA; 4 Department of Biology, Washington University, St. Louis, Mo, USA

## Abstract

**Background:**

The question of a genetic contribution to the higher prevalence and incidence of end stage kidney disease (ESKD) among African Americans (AA) remained unresolved, until recent findings using admixture mapping pointed to the association of a genomic locus on chromosome 22 with this disease phenotype. In the current study we utilize this example to demonstrate the utility of applying a multi-step admixture mapping approach.

**Methods:**

A multi-step case only admixture mapping study, consisted of the following steps was designed: 1) Assembly of the sample dataset (ESKD AA); 2) Design of the estimated mutual information ancestry informative markers (n = 2016) screening panel 3); Genotyping the sample set whose size was determined by a power analysis (n = 576) appropriate for the initial screening panel; 4) Inference of local ancestry for each individual and identification of regions with increased AA ancestry using two different ancestry inference statistical approaches; 5) Enrichment of the initial screening panel; 6) Power analysis of the enriched panel 7) Genotyping of additional samples. 8) Re-analysis of the genotyping results to identify a genetic risk locus.

**Results:**

The initial screening phase yielded a significant peak using the ADMIXMAP ancestry inference program applying case only statistics. Subgroup analysis of 299 ESKD patients with no history of diabetes yielded peaks using both the ANCESTRYMAP and ADMIXMAP ancestry inference programs. The significant peak was found on chromosome 22. Genotyping of additional ancestry informative markers on chromosome 22 that took into account linkage disequilibrium in the ancestral populations, and the addition of samples increased the statistical significance of the finding.

**Conclusions:**

A multi-step admixture mapping analysis of AA ESKD patients replicated the finding of a candidate risk locus on chromosome 22, contributing to the heightened susceptibility of African Americans to develop non-diabetic ESKD, and underscores the importance of using mutual information and multiple ancestry inference approaches to achieve a robust analysis, using relatively small datasets of "affected" only individuals. The current study suggests solutions to some limitations of existing admixture mapping methodologies, such as considerations regarding the distribution of ancestry information along the genome and its effects on power calculations and sample size.

## Background

Chronic kidney disease (CKD) encompasses a spectrum of different pathophysiologic processes associated with abnormal kidney function, and a progressive decline in glomerular filtration rate (GFR), culminating in a degree of irreversible loss of GFR (reduction to < 15 ml/min per 1.73 m^2^) necessitating renal replacement therapy (dialysis or transplantation) in order to sustain life (Stage V Chronic Kidney Disease, also known as End Stage Kidney Disease or ESKD). Even in those constituencies where such renal replacement modalities are available, quality of life and lifespan are dramatically reduced, and in many regions of the world where renal replacement therapy is not available, ESKD is fatal [1].

It has long been recognized that there are striking ethnic differences in the incidence and prevalence rates of ESKD [2]. In the United States (US) African Americans (AA), have the highest incidence and prevalence of ESKD [3]. Previous studies have concluded that the higher prevalence of both diabetes and hypertension, which have been considered the two leading etiologies of CKD, among AA are not sufficient to explain this increased risk [4,5]. Other studies have indeed questioned whether chronic hypertension is a cause of CKD in AA, or rather a consequence of other forms of primary kidney disease [6]. Other epidemiologic studies have concluded that differences in conventional clinical, socio-demographic, or lifestyle factors, although important, are insufficient to account satisfactorily for the excess risk of ESKD among AA [7,8]. Adjustment for the various risk factors associated with kidney disease led to an estimated 1.87 relative risk of ESKD among AA compared to Caucasian males [7]. ESKD is most probably a multi-factorial disease phenotype affected by both genetic and environmental process. Moreover, with respect to the genetic contribution, as is the case for many common complex adult onset disorders, it is likely that common variants with variable penetrance at multiple genetic loci, each with modest contribution, might contribute to the disease phenotype and susceptibility to ESKD [9]. In previous studies, polymorphisms in several genes such as the plasma kallikrein gene and the human homologue of the rodent renal failure 1 gene have demonstrated both linkage and association with ESKD in the AA population [10-12]. Most strikingly, two studies [13,14] reported that the genetic locus *MYH9 *on chromosome 22, explains much of the increase in non-diabetic ESKD among AA, but made no apparent contribution to the increased susceptibility of AA to ESKD related to diabetic kidney disease.

Both of these previous studies utilized admixture mapping, (AM), as the population-based genome wide mapping tool. AM makes use of the relatively recent admixture in the Americas of individuals of African and European ancestry [15,16], and is based on the assumption that the low rates of gene flow prior to admixture of the two ancestral populations generated pre-admixture allele frequency differences at many loci across the entire genome [17]. When two such previously genetically differentiated populations admix, extensive gamete phase imbalance or linkage disequilibrium is generated in the admixed population. The gene flow that takes place during admixture results in the temporary generation of long stretches of DNA sequence (haplotype blocks) in which polymorphic loci are in admixture linkage disequilibrium. The AM method is applied in three steps. First, either cases, or both cases and controls are genotyped via a panel of ancestry informative markers (AIMs). Second, the ancestry of each sampled individual is inferred along the entire genome. Finally, the genome of affected individuals is scanned in search for a region that shows an elevated frequency of the ancestry with a higher risk for the studied phenotype. AM has a statistical power that is similar to association mapping to detect disease-associated variants that differ markedly in frequency between populations [18]. In AM, the ability to detect haplotypes that contain a disease-associated variant for a specific complex disease is maximized by analyzing the markers that are most divergent between the ancestral populations of the admixed group, namely Ancestry Informative Markers (AIMs).

Despite the reproducibility of the locus on chromosome 22 as a candidate region for the increased risk of African Americans (Nelson et. al 2010), and Hispanic Americans (Behar et al 2010) to develop ESKD, the causative mutation accounting for the AM signal was not identified within the MYH9 gene (Nelson et al 2010). In the current study we utilize an information-theory based measure which we have recently developed [19], designated as "expected mutual information" (EMI) and apply a simple and effective algorithm for the selection of panels that strives to maximize the EMI score. EMI computes the impact of a set of markers on the ability to infer ancestry at each chromosomal location. Using these tools we present here an hierarchical multi-step AM approach which validates the previously reported chromosome 22 locus associated with increased risk for non-diabetic ESKD among AA, and propose that this approach can be applied more widely to extend AM to other complex disease phenotypes with moderate differences in prevalence and incidence rates between ancestral populations, even with small to moderate size samples sets of (fewer than several hundred individuals) of affected only individuals.

## Methods

### Study Design

A multi-step case only AM study was designed, suitable to the available sample set and genomic association question. Study design consisted of the following steps: 1) Assembly of the sample dataset; 2) Design of the AIMs screening panel 3); Genotyping a sample set whose size is determined by a power analysis appropriate for the initial screening panel of genome wide ancestry informative SNP markers; 4) Inference of local ancestry for each individual and identification of regions with increased AA ancestry using two different ancestry inference statistical approaches; 5) Enrichment of the initial screening panel with additional AIMs markers located on the chromosome of interest identified in step 4; 6) Power analysis of the directed enriched panel 7) Genotyping of additional samples using the enriched panel. 8) Re-analysis of the genotyping results to identify a genetic risk locus.

#### 1) Assembly of the sample dataset

The patient group included all consenting ESKD patients recruited from DaVita Cabrini and Fresenius dialysis facilities in New York (NY) City. Informed consent was obtained from all patients and a questionnaire completed. The entire clinical research protocol was approved by the IRB of the participating institutions, Ancestry was designated by self identification, the birthplaces of each subject and both parents were also recorded. Some of the samples in the current study, were also included as part of a comparative analysis in a study which reported the role of MYH9 alleles in the risk for ESKD among Hispanic Americans [20]. Self identified ancestry was shown in previous studies to be a reliable measure of ancestry [21]. Detailed characteristics of the cohort population are provided in Table 1. For the screening genotyping step the inclusion criteria were: self identified AA ESKD patients, with place of birth of the subject and both parents in the U.S. Individuals with the following diagnoses were excluded: etiology of ESKD due to polycystic kidney disease, obstructive uropathy, amyloidosis, cancer related renal failure. The etiology of renal disease was obtained from the patients' medical records as recorded in the hemodialysis facilities. Diabetes related ESKD was defined as self-reported and/or prevalent treatment with insulin and/or oral hypoglycemic agents or as documented in the written medical record. Age of onset of first dialysis was also obtained from the medical record. Additional samples for the enrichment step were randomly chosen self identified AA ESKD patients who also met foregoing inclusion and exclusion criteria.

**Table 1:** Clinical Characteristics of the cohort population.

	Screening Panel	Enrichment Panel	Total
Males	282 (49%)	151	433

Females	294 (51%)	106	400

Age	60 ± 13	61 ± 13	61 ± 13

Years on Hemodialysis	4.2 ± 3.9	4.3 ± 3.8	4.3 ± 3.9

Diabetes	277 (48%)	155 (60%)	432

No diabetes	299 (52%)	102 (40%)	401

Total	576	257	833

#### 2) Design of the AIMs screening panel

As a platform for the AIMs screening panel we used a panel of 2000 AIMs validated by Tian et al [22] (Tian Panel). In order to accommodate this panel to the Illumina GoldenGate bead array technology a validation process for the Illumina GoldenGate assay was utilized, in which 1600 AIMs were predicted to yield high genotyping rates. In order to enrich this 1600 AIMs panel we developed a novel computational platform, for selection of ancestry informative markers, using an information-theory based measure, called Expected Mutual Information (EMI), as previously reported [19]. EMI computes the impact of a set of markers on the ability to infer ancestry at each chromosomal location. We further developed a simple and effective algorithm for the selection of panels that strives to maximize the EMI score [19]. Ancestral population allele counts for admixture analysis were compiled from HapMap [23]. Using the EMI based enrichment approach we validated an additional 416 Illumina GoldenGate AIMs markers, thus generating the 2016 AIMs screening panel, (Additional File 1). All physical positions and SNPs details reported in the current study were based on NCBI genome build 36, and dbSNP build 127.

#### 3) Power analysis and genotyping

In order to determine the sample size needed for the case only AM screening step we conducted a set of simulations using the EMI based screening panel, using ANCESTRYMAP, and compared its performance to the Tian panel.

A multiplicative risk model parameterized by several ethnicity relative risk values (ERR) was used. We generated samples of admixed-individual genotypes for a case only analysis using ANCESTRYMAP. For each run, a single marker location on chromosome 4 and 22 was designated as the disease-predisposition locus. In order to evaluate the performance of the EMI based screening panel across the entire chromosome, a set of disease-predisposition loci were chosen using a resolution of four markers per Cm; consequently, 627 and 186 uniformly selected locations across chromosome 4 and 22 (respectively) were used in the power experiments. A range of ethnicity relative risk (ERR) ratios, between 0.4 and .0.8, were set as the disease model parameters, all assuming that the African population exhibits the higher disease risk. Power was measured as the proportion of runs which identified the putative disease loci. We have tested the power to detect a disease loci with genome log-factor >2 using ANCESTRYMAP. The power of the current screening panel was compared to the 2000 AIMs panel which appeared in Tian et.al [22], and p values were calculated using the Chi square test.

According to the power simulations for an ethnicity risk ratio of 0.6 only 576 AA ESKD patients were needed in order to reach a power of 80%, using the EMI based screening panel, and assuming a homogeneous phenotype in terms of the risk locus. Accordingly 576 self declared AA ESKD patients were genotyped for the 2016 AIMs screening panel using the GoldenGate assay. Only SNPs with genotype call rates of 85% or more were included in the analysis phase.

#### 4) Inference of local ancestry for each individual and identification of regions with increased African ancestry using two different ancestry inference statistical approaches

Both ANCESTRYMAP [24] and ADMIXMAP [25] programs were used to assess individual ancestry proportions and to scan the genome for regions of African ancestry that differ significantly from the genome average. For ANCESTRYMAP, risk models ranging from 0.25- to 4-fold risk per African chromosome were assessed. We evaluated significance by LGS scores reported by ANCESTRYMAP. ANCESTRYMAP was used with the parameters of 100 for burn-in and 200 for follow-on iterations for all Markov chain-Monte Carlo runs as recommended. ANCESTRYMAP also calculated a LOD score for genome-wide significance; a score greater than 1.0 was considered as a candidate region and a score greater than 2.0 was considered significant [24].

ADMIXMAP: 500 iterations were used for burn-in of the Markov chain. The tests for linkage provided in ADMIXMAP [25] are score tests based on the missing-data likelihood. *U *is evaluated as the posterior expectation of the realized score, and the observed information *V *is calculated by subtracting the missing information (posterior variance of the realized score) from the complete information (posterior expectation of the realized information). The models from ADMIXMAP calculate results in terms of standard normal Z statistics and p values. In this study we report the minus log base 10 of the affected only p values from ADMIXMAP final report.

#### 5) Directed enrichment of the screening panel

The Results of step 5 were reviewed, and regions for further investigation were chosen according the following criteria: 1) LGS Local ≥ 2: The log likelihood of the LGS score obtained by averaging over all the markers on a chromosome as computed by ANCESTRYMAP. 2) In ADMIXMAP, p value ≤ 10^-3 ^for at least 2 consecutive AIMs within a range of 4 Cm.

Chromosomes with screening AM loci which met the foregoing criteria were further genotyped using an enriched AIMs panel, selected using the EMI algorithm. As indicated in the Results section - the chromosome of interest to which the AIMs enrichment markers were added is chromosome 22, based on the analysis of the genome wide screening marker panel. The enrichment process excluded AIMs that had already been genotyped in the non-enriched EMI panel. A total of an additional 39 AIMs were available using this approach for chromosome 22 to generate the enriched AIMs marker panel (Additional File 2). All physical positions and SNPs details reported in the current study were based on NCBI genome build 36, and dbSNP build 127.

#### 6) Power Analysis of the directed enriched panel

The same methodology for power analysis as step 3 was used in order to assess the power and sample size needed in order to validate the chromosome 22 preliminary signal. In the second step power analysis we have used the enriched screening panel (78 AIMs on chromosome 22) together with the AIMs of chromosome 1 in order to evaluate individual ancestry more accurately. Accordingly for an ethnicity risk ratio of 0.6 an additional 240 AA ESKD patients were needed in order to reach a power of 80%, using the EMI based enriched panel for chromosome 22. It should be noted that at the time of the second step power analysis, the heterogeneity of the sample set into two disease categories (diabetic and non-diabetic) was not yet known and therefore was not included in the power analysis.

#### 7) Genotyping of additional samples using the directed enriched panel

An additional 257 Self identified AAs with ESKD (155 with diabetes and 102 without diabetes) were genotyped using the directed enriched panel together with the 576 samples, which had undergone genome wide genotyping.

#### 8) Re-analysis of the genotyping results to identify a genetic risk locus

The results were reanalyzed using both ADMIXMAP and ANCESTRYMAP, exactly as described for Step 5 above. A statistically significant locus was considered as a locus with LGS >5 using ANCESTRYMAP, and a - LOG (P) > 5 using ADMIXMAP for at least two consecutive SNPs. In order to take into consideration the effect of LD among the ancestral populations on the results, AIMs which were 50 kB or less close to each other were omitted (Additional File 2).

## Results

### Power of the screening panel

**Figure 1:**
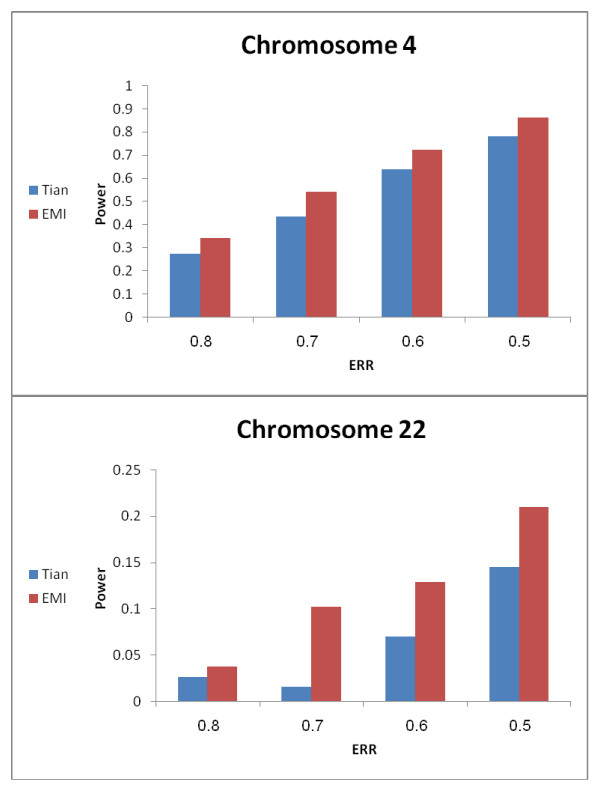
**Power analysis of the screening panel**. For the power analysis of the screening panel, admixed-individual genotypes for a case only analysis using ANCESTRYMAP were simulated. A set of disease-predisposition loci were chosen using a resolution of four markers per Cm. A range of ethnicity relative risk (ERR) ratios, between 0.4 and .0.8, were set as the disease model parameters. Power was measured as the proportion of ANCESTRYMAP runs which identified the putative disease loci with genome log-factor >2. The power of the screening panel was compared to the 2000 AIMs panel which appeared in Tian et.al [22].

We first sought to assess the added power conferred by addition of EMI markers to an existing AM single nucleotide polymorphism (SNP) panel [22], using markers on two chromosomes of different length and with differing marker density in previously published and available AIMs panels - namely, chromosomes 4 and 22. Such power analysis for chromosome 4 shows that for a sample set of 576 ESKD patients, and an ethnicity relative risk (ERR) of 0.5 using the EMI enriched screening panel yielded a power of 0.86 in comparison to 0.78 using the non-enriched panel (p < 0.0001). For other ERRs models, our analysis showed that the EMI enriched screening panel had significantly higher statistical power in comparison to a screening panel with similar AIMs density. The results for chromosome 22 demonstrate the same trend, albeit with a reduced power (Figure 1a).

An ANOVA analysis of the Fisher information content (FIC), as provided in Tian et al [22] for the entire panel of 2000 SNPs therein demonstrated variability in the information content for the various chromosomes (Additional File 3). Accordingly, we compared the distribution of FIC and EMI scores over the entire EMI enriched screening panel for all chromosomes (Additional File 4). The lowest mean EMI and FIC scores were observed for chromosomes 6, 18, 19, and 22, and we further observed variability at chromosome ends. We then calculated the probability for distribution differences among FIC values for various chromosomes using the Kolomogorov-Smirnov test. The results for chromosome 22 (Additional File 5) demonstrated a significant difference in the distribution of FIC values for chromosome 22 in comparison to chromosomes:1,4,9,13,14,15,20, and X. Chromosomes 18 and 19 showed even more significant distribution differences in comparison to most chromosomes (data not shown). Chromosome 13, 20 and X showed significantly higher mean EMI and FIC score values, in comparison to other chromosomes (Supp. Figure1). X chromosome variation marker analysis, generally shows greater differentiation among human populations than autosomes, related to the difference in effective population size, and as reflected by gene flow and or drift patterns [26]. Inclusion of X chromosome markers, can possibly introduce a confounding effect of differential gender proportion in the founding and the admixed populations. Therefore, the results of X chromosome admixture mapping are not included in the current study.

Since previous studies [19,22,27] have demonstrated a correlation between informativeness of SNPs and the power to detect susceptibility loci using AM, the variability in FIC and EMI score distribution along the genome might explain the large differences in ancestry inference power among chromosomal loci - such as observed for chromosomes 4 and 22.

### Screening panel results

**Figure 2:**
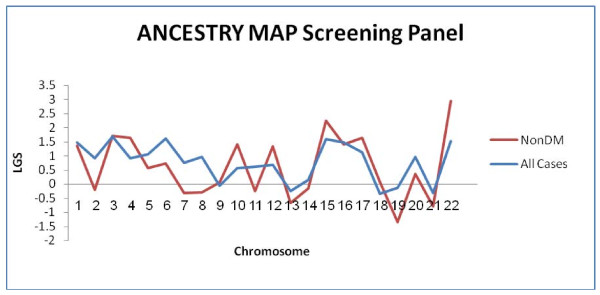
**Locus genome statistics (LGS) local of the screening panel**. The locus genome statistics (LGS) local as provided by ANCESTRYMAP for each chromosome using the screening panel (2016 AIMs) and a total of 576 ESKD AA patients (Blue line) of whom 299 ESKD patients did not have diabetes mellitus (NonDM) (Red line).

Following the hierarchical approach outlined in Methods - we first applied AM analysis using the screening panel, to the entire sample set (576 ESKD AAs) using ANCESTRYMAP. No candidate loci were observed according to the stepwise criteria for proceeding to peak choice, as outlined in Methods, namely a locus genome statistic (LGS) local ≥ 2 (Figure 2). In contrast, three chromosomal candidate loci were observed according to the criteria of a p value ≤ 10^-3 ^for at least 2 consecutive AIMs in a range of 4 centimorgans (Cm) using ADMIXMAP - namely chromosomes 5 and 22 (Table 2). Next, we repeated the analysis using both ANCESTRYMAP and ADMIXMAP after subdividing the sample set based on reported aetiology of ESKD into diabetic (n = 277) and non-diabetic (n = 299) categories (Table 1). Despite the reduction in sample size resulting from this subdivision, ANCESTRYMAP revealed peaks located on two chromosomes (15 and 22) (Figure 2). Analysis of this same non-diabetic sample subset using ADMIXMAP, revealed peaks located only on chromosome 22. Thus only chromosome 22 demonstrated a peak for the non-diabetic subset using both analytic approaches (see step 5 in Methods stepwise criteria). No peaks were evident for the n = 277 diabetic samples, using either ANCESTRYMAP or ADMIXMAP.

**Table 2:** LOG (P) score of candidate loci by ADMIXMAP screening panel.

**Locus Name**	**Position (Cm) Build 36**	**Position (Bp) Build 36**	**Chromosome**	**- LOG (P) All Cases**	**- LOG (P) NonDM**
rs6811604	50.57	29549192	4	3.32	0.37

rs3923114	76.42	68362054	5	3.05	0.34

rs7700504	81.30	73347096	5	3.15	0.46

rs16872999	83.74	75235507	5	3.38	0.81

rs7712675	85.80	76174323	5	3.06	0.72

rs12938039	94.49	64732231	17	3.05	0.01

rs7286127	37.43	34828811	22	3.77	3.95

rs5756133	38.16	35023926	22	3.46	3.95

All AIMs with a - LOG (P) greater than 3 are presented, for the all ESKD patients (576 samples) and for the non diabetic only patients (299 samples) Cm - centimorgans. Bp - base pairs.

**Figure 3:**
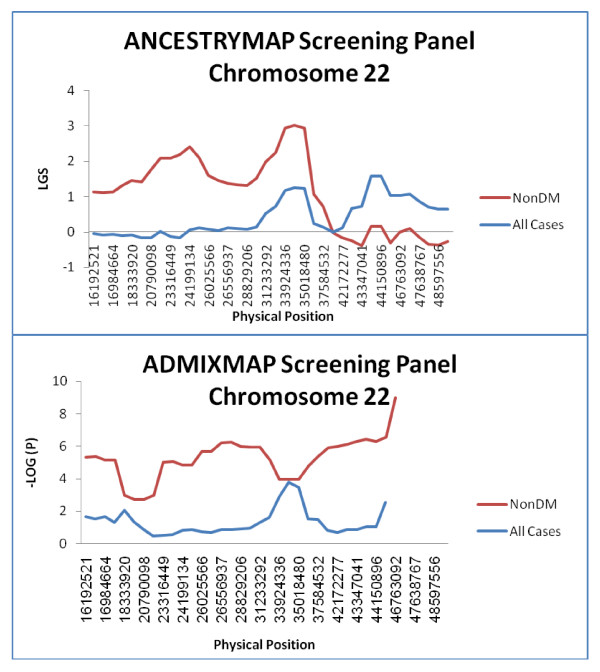
**Results of the screening panel for chromosome 22 by ANCESTRYMAP and ADMIXMAP**. LGS and - minus LOG Base10(P) are provided by ANCESTRYMAP and ADMIXMAP respectivly for various AIMs along chromosome 22. Altogether 39 AIMs on chromosome 22 were genotyped using the screening panel and a total of 576 ESKD AA patients (Red line) were genotyped, of whom 299 ESKD patients did not have diabetes mellitus (NonDM) (Blue line).

While the screening panel, revealed peaks on chromosome 22 using both ANCESTRYMAP and ADMIXMAP, it should be noted that these chromosome 22 peaks did not reach significance thresholds for disease risk association (Figure 3) for the sample sets utilized, thus prompting enrichment of the panel as the next step.

### Enrichment of the screening panel

39 additional SNPs (chosen based on the EMI algorithm [19]) on chromosome 22 (Sup. Table 1) were added to the screening panel, and are designated as the enriched panel. Furthermore, since the screening panel results revealed non-significant peaks only for the relatively small non-diabetic subset, and since power analysis indicated the need for a greater samples size, an additional 102 non-diabetic samples were added (Table 1).

**Figure 4:**
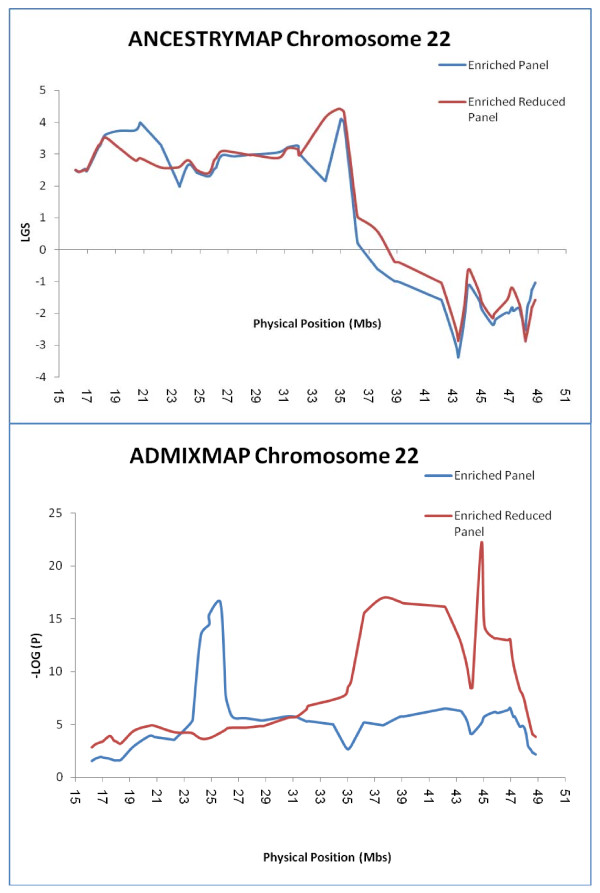
**Results of the enriched panel by ANCESTRYMAP and ADMIXMAP for chromosome 22**. LGS and minus LOG Base10(P)are provided by ANCESTRYMAP and ADMIXMAP respectivly for various AIMs along chromosome 22, using the enriched panel (78 AIMs), (Blue line). The results of the enriched reduced panel (67 AIMs), (Red line) represent the LGS and - LOG (P) after the omission of AIMs which were sequentially spaced at < 50 kB.

As shown in Figure 4 and Supp. Table 2, analysis using ADMIXMAP revealed the significant peaks (- LOG (P) > 5) at several loci along the chromosome, including the region between rs7285549 to rs2213758 (- LOG (P) > 10) and the region between rs3752596 to rs2076084. In contrast, the LGS scores for the peaks obtained using ANCESTRYMAP at corresponding regions rs165824 to rs8138979 and rs3752596 to rs2076084 (Figure 4) did not exceed LGS > 5. Previous analyses have indicated that spurious peaks can arise in linkage and association studies, including admixture mapping and can be attributed to LD effects in ancestral populations. Therefore we reasoned the high density of markers following enrichment on chromosome 22 should be addressed by utilizing markers limited to an adjacent spacing of >50 Kb. Analysis using ADMIXMAP yielded a - LOG (P) score of ~15-16 spanning 7 consecutive SNPs in the region of rs4821667 to rs739016 (Supp. Table 2) (Figure 4). Notably, this region contains the MYH9 gene, as well as the neighbouring APOL1 gene, more recently shown to contain coding region variants which are candidates for ESKD risk causation [28,29]. Analysis using ANCESTRYMAP yielded a maximum LGS score of 4.428 at SNP rs7286127, whose physical location is 34.8 Mbs, and overlapping the set of significant SNPs obtained using ADMIXMAP (Figure 4).

The excess of African ancestry associated with the genomic region on chromosome 22 noted above, is also presented in Table 3. Furthermore the genome wide score for disease risk attributed to having one African ancestry allele is 1.6 and for African ancestry alleles the risk is 2.6.

**Table 3:** Excess of African ancestry for markers in risk region compared to genome average in African American ESKD subjects.

Locus Name	Expected African Ancestry Among AA*	Estimated African Ancestry Among AA ESKD patients**	% Change in African Ancestry
rs7286127	**0.833**	**0.923**	**10.8%**

rs5756133	**0.865**	**0.954**	**10.2%**

*Expected loci specific African ancestry was calculated from the African allele frequency as was reported by dbSNP build 127, multiplied by genome average African ancestry as was reported by ANCESTRYMAP.

**Estimated African ancestry was reported for each locus using ANCESTRYMAP.

## Discussion

The AM analysis in the current study confirms the results of previous studies [13,14] which identified a genomic region on chromosome 22 conferring increased genetic risk for AA to develop non-diabetic ESKD. This finding was recently extended to Hispanic Americans, whose risk for ESKD is intermediate between that of AA and Europeans, and further higher association SNPs within MYH9 were identified, and proposed to narrow the region containing a putative disease phenotype causal mutation initially pointing to the MYH9 gene [20,30]. Since these initial reports, the gene now thought to contain the functional causative mutation accounting for the AM peak is the neighboring APOL1 gene, encoding the apolipoprotein L-1 protein, with the MYH9 marker associations being the result of very high levels of linkage disequilibrium, powerful evolutionary selection and hitchhiking of the MYH9 variants with putative APOL1 causative mutation [28,29].

In contrast to the two previously reported AM studies, our results were obtained using a different and independent marker panel, a different and independent patient sample set, and two different analytic approaches. The importance of replicating identification of genetic risk loci for common complex disease susceptibility has been highlighted as a *sine qua non *for proceeding with further mechanistic research for such a locus, and certainly for genetic counseling and clinical applications. In the case of AM in particular, it has been recommended that data should be analyzed using at least two of the several different available ancestry inference programs [18]. Failure to replicate has been a common feature in the search for common variants of candidate genes affecting the risk of complex diseases [31]. In the case of ESKD among AA, a long standing controversy existed as to the relative contribution of societal and demographic as opposed to genetic factors in the increased risk for ESKD [32,33]. Furthermore other rationales concerning the etiology of the higher prevalence of ESKD have been proposed, such as lower mortality rates of AA ESKD patients [34]. The current study represents the third AM study confirming the presence of a genetic susceptibility locus for ESKD in African Americans- and also confirms that the increased risk conferred by this locus seems to be restricted to non-diabetic ESKD. In the sub-group of non-diabetic ESKD AA, the increased genetic risk conferred by the African ancestry risk allele explains the majority of the increased risk (1.6 for heterozygote and 1.87 increased risk in the epidemiological studies[7]). Though the locus susceptibility was reproducible in all 3 studies, the exact location of the risk causative mutation(s) cannot be inferred from AM studies alone. Indeed, even the initial candidate gene (MYH9) implicated in the first two AM studies, has since been superseded by a more statistically and biologically plausible gene (APOL1), though it has been the AM approaches which focused attention to the chromosome 22 region containing these genes in the first place[13,14].

Each of the three studies used a different sample set, but all of the samples were of African American heritage. The current study also utilized a different AIMs panel and two different analytical methods, confirming the utility of AM as an effective mapping approach using relevant populations and disease phenotypes. The use of two different analytic approaches also reduces the risk of false positive results inherent in the methodology of AM and emanating from the complex patterns of LD among panel SNPs in ancestral populations. Future research will be needed in order to validate the genetic risk conferred by the chromosome 22 loci in other populations, other stages of CKD, and possibly other modifier loci, taking into the account the complex heritability and multi-factorial nature of ESKD[35,36]

The current study also highlights additional points of more general interest pertaining to AM which might be of interest and utility in application to other sample sets and disease states - especially when only small sample sets or weaker ERRs are involved. Examples could include the increased susceptibility to systemic lupus erythematosus (SLE) [37] and the higher incidence of lupus nephritis among AA SLE patients [38,39], or diseases with reduced prevalence among AA (e.g. HCV clearance)[40] and psoriasis[41]. In particular, we propose use of a stepwise hierarchical approach and novel ancestry panel tools in AM analysis projects where ERR or sample size may be limiting. Furthermore, we have used a case only study design, which for a given sample size and marker set, has been suggested by others to be more powerful than a case control study design (reviewed in Montana et.al [42]). However, it should be noted that such a case only study design, might be more sensitive to inaccuracies in estimation of specified allele frequencies. This is less problematic with current builds of dbSNP, which are based on larger sample sets in comparison to the SNPs datasets that were available in earlier admixture mapping studies. Notwithstanding these reservations, the analysis based on real samples in the current study, in which the admixture peak and its genetic loci have been validated in independent studies, lends further confidence to the predicted power and accuracy of an appropriately formulated case only study design.

The results of the power analysis of the non-enriched screening panel in the current study, demonstrated a non-homogenous distribution of ancestry information across the genome (Additional File 3). The main differences were observed while inspecting the distribution of ancestry information along several chromosomes, which exhibited marked differences in the information content among and along the lengths of chromosomes, generating a situation in which the mean information content is quite stable over the genome as a whole, but with marked regional differences in the distribution of information content in comparison to the genome as a whole. This was the case for chromosomes 18, 19 and 22 - the latter of which turned out to be the chromosome harbouring the genetic susceptibility locus of interest. While fortuitous - this turned out to be informative in the formulation of an effective step wise approach of potential general utility. Thus the heterogeneity of information content, involving chromosome 22, likely explains the differences in the power of the non-enriched screening panel to identify true positive loci along chromosome 22 (Figure 1). Presumably the regions on chromosome 22 with lower information content yield lower accuracy in the ancestry inference and therefore reduced the capacity to identify a disease risk locus in the power experiments, which were conducted systematically over all of the chromosomes. This would be expected to yield a false negative result for a true disease risk locus on chromosome 22, as was indeed the case for the actual sample set analysed - which yielded suggestive peaks according to pre-set criteria as outlined in Methods, but peaks which did not meet rigorous association significance scores. Having achieved the suggestive peaks - this limitation was then overcome using both the EMI enrichment process which strives to maximize the chromosome-wide information by taking into account mutual marker information, together with the power enhancement afforded by the enriched panel and adjustment of the sample size accordingly.

Since the information content is not homogeneously distributed over the chromosomes, the relative risk conferred by an associated locus is not known in advance, leading to the expectation of a shift in the estimated power. Therefore, we suggest that following the addition of AIMs, re-analysis of power should be undertaken in order to estimate the number of samples needed for a locus of interest which has arisen in a screening panel. It should be noted that this hierarchical approach, using an initial genome wide screening panel - could potentially overlook candidate peaks (type II error), which would then not make it to the step of scrutiny by an enriched panel on the chromosome or region of interest. It is reassuring that in the current study - an initial screening panel of only 2,016 SNPs and what turned out to be an initial sample set of only 277 relevant (non-diabetic ESKD) samples, led to the appropriate next steps, using ANCESTRYMAP and ADMIXMAP as the initial ancestry inference programs, which converged on one shared risk locus.

An additional important lesson from the current study is the inferential power achieved by combining two different analytic and computational platforms, once genotype information is available. Although previous guidelines for AM methodology recommended the use of at least 2 analytical approaches [18], most published AM studies have generally used only one statistical analysis platform [13,14,43-48]. The study by Nalls et.al used all three currently available statistical programs (ADMIXMAP, STRUCTURE, and ANCESTRYMAP), and concluded that it was difficult to directly compare the significance values from the three programs, because each program calculates a different statistic for association [49]. In the current study we followed this recommendation and used both ANCESTRYMAP and ADMIXMAP as statistical platforms. Tian et.al [22] had previously reported a high rate of false positive results when using ANCESTRYMAP at chromosomal positions in complete LD. In contrast, no false-positive peaks were observed in the same simulations using ADMIXMAP. Our goal in the screening panel step of the current study was to identify one or more candidate true positive regions containing a common risk variant.

ANCESTRYMAP was used because of its unique LGS local score, which can point to a possible association for a given chromosome, unlike ADMIXMAP software which instead provides statistics for individual markers only. Indeed, in the current study, ANCESTRYMAP yielded no peaks across all chromosomes for the entire sample set, but did yield a candidate peak in the non-diabetic subset of samples (Figure 2, 3), consistent with the previous reports by Kopp et.et [14] and Kao et.al [13]. A second peak on chromosome 15 was also evident. The second peak in ANCESTRYMAP on chromosome 15 was not observed using ADMIXMAP (Additional File 6). Only chromosome 22 yielded a candidate locus using both ANCESTRYMAP and ADMIXMAP. It was this finding which then prompted the next enrichment step directed to this locus.

The goals of the enrichment process were to validate and fine tune the screening panel results, and to narrow the region. The results of ANCESTRYMAP with its LGS local parameter yielded two candidate peaks on chromosome 22 (Figure 4). Based on the simulation by Tian et. al [22] we considered the possibility that one of these peaks might be a false positive result, consequent to the effect of very high levels of LD in the ancestral populations. Indeed using ADMIXMAP, it was clearly demonstrated that the deliberate omission of SNPs with the highest D' values in the ancestral populations pointed only to the peak at nucleotide positions 35-37 Mbs, and giving a significant association (- LOG (P) > 10) (Figure 4). This result highlights the importance of spacing SNPs in such a way as to avoid spurious peaks presumed due to LD interference, which in the case of the current study required spacing of SNPs at distances greater than 50 kB. Tian et al [22] used simulations to suggest that residual LD, even at spacing of 100 kB as in the screening panel, can yield spurious peaks, using a case only design and ANCESTRYMAP, but not ADMIXMAP as the statistical analysis approach. Using experimental data, and both a screening and enriched panel, we have been able to verify this effect of residual LD, which occurs using either ANCESTRYMAP or ADMIXMAP - but which can be overcome by addition of appropriately spaced markers so as to avoid presumed LD interference. It is evident from the current study that use of ADMIXMAP alone, will not overcome this effect, as had been suggested based on simulation studies [22]. Our results support previous observations that residual LD in the ancestral populations causes false positive signals[50], thus limiting the density of AIMs that can be used and highlighting the utility of using both multiple analytic approaches, as well as selecting carefully spaced enrichment markers, especially for moderate risk loci. We can recommend 50 kB as an empirical lower limit to spacing of AIMS in AM, with judicious use of more than on analytic approach and choice of coinciding peaks for the further detailed search for candidate genes.

In the current study it may be observed that the p values obtained from ADMIXMAP, were more significant in comparison to those obtained using the ANCESTRYMAP LOD scores (Figure 4 reduced panel). One possible explanation is that ADMIXMAP does not apply any correction for multiple hypothesis testing [42]. Furthermore as can be observed in Figure 4 ADMIXMAP results are greatly influenced by the omission of dense SNPs (with high LD in ancestral populations), while the corresponding changes for ANCESTRYMAP are only moderate. However, we caution that while highly tenable, we cannot conclude definitively that this is the source of discordance between ANCESTRY MAP and ADMIXMAP, since each of these analytical programs calculates a different statistic for LD.

## Conclusions

A multi-step AM analysis of ESKD patients, using a new patient sample set, and a different SNP marker panel, replicates the previously reported identification of a genetic disease phenotype risk locus located on chromosome 22, which contributes to the increased risk for non-diabetic ESKD in AA. In addition, the current study stresses the importance of using two different statistical approaches for the analysis of AM. This study also highlights the importance of evaluating LD resulting from marker density - an important factor which yields inflation in significance, which was has also been observed in other type of linkage and association analysis [50]. An interesting observation to be further studied is the non-homogeneous distribution of ancestry information across the genome and its possible practical utility in terms of power analysis and AM experiment design.

## Competing interests

The authors declare that they have no competing interests.

## Authors' contributions

Contribution: LIS designed the study, performed the majority of the research, analyzed the data and wrote the manuscript; SB designed the study and performed the analysis; WGW collected the sample set and contributed to study design GY performed the research; AT designed the study; DG designed the study, and participated in writing the manuscript. KS conceived the idea of the study, participated in study design and writing of the manuscript. All authors read and approved the final manuscript.

## Pre-publication history

The pre-publication history for this paper can be accessed here:

http://www.biomedcentral.com/1755-8794/3/47/prepub

## Supplementary Material

Additional file 1 **Expected Mutual Information (EMI) ancestry informative markers screening panel**. The AIMs were chosen according to EMI enrichment algorithm. 2016 markers were chosen. Click here for file

Additional file 2 **Directed enriched panel chromosome 22**. Enrichment of the screening panel was applied to chromosome 22. All additional AIMs were chosen according to EMI algorithm. 39 additional AIMs were added. For the sub-analysis of the effect of ancestral LD markers which were 50 kB apart from each other or less were omitted from the analysis. In the status column 1 = AIMs from screening panel 2 = AIMs from directed enriched panel. 3 = AIMs from directed enriched panel which were omiited due to a distance of less than 50 kB from other markers. Click here for file

Additional file 3 **Distribution of Fisher Information Content (FIC) along the genome for the 2016 markers of the screening panel**. For each chromosome the mean FIC (2 red circles), two standard deviations of the FIC value (dashed line) and outliers of AIMs with high FIC (red plus) are presented. Click here for file

Additional file 4 **Distribution of expected mutual information (EMI) score along the genome for the 2016 markers of the screening panel**. The Y axis for each chromosome represents the EMI score (the information of a set of markers regarding the ancestry of a chromosomal position, the X axis represents the genetic position of all AIMs in the screening panel in Cm according to the Marshfield genetic map. Click here for file

Additional file 5 **Differences in the distribution of FIC values between chromosome 22 and other chromosomes**. For each chromosome the mean FIC (circles) and the range of FIC (horizontal lines) are displayed. P values represent differences in the distribution of FIC as was calculated by pairwise Kolmogorov-Smirnov test. Click here for file

Additional file 6 **Chromosome 15 LGS and - LOG (P) scores of the screening panel by ANCESTRYMAP and ADMIXMAP respectively**. LGS and - LOG (P) are provided by ANCESTRYMAP and ADMIXMAP respectivly for AIMs along chromosome 15. Using the screening panel a total of 576 ESKD AA patients (Red line) were genotyped of whom 299 ESKD patients did not have diabetes mellitus (NonDM) (Blue line). Click here for file
