# Supplementary figures and images for: Admixture mapping of end stage kidney disease genetic susceptibility using estimated mutual information ancestry informative markers
Source: BMC Med Genomics. 2010 Oct 18;3:47. doi: 10.1186/1755-8794-3-47 (PMC2975638; doi:10.1186/1755-8794-3-47)

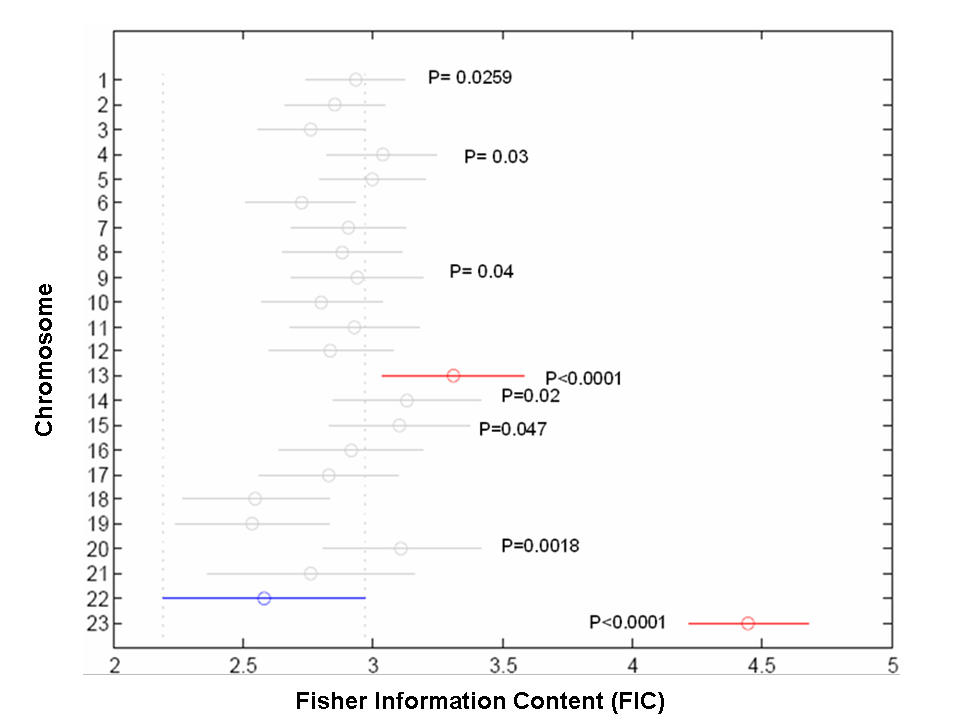

Supplement: Additional file 5 — Differences in the distribution of FIC values between chromosome 22 and other chromosomes. For each chromosome the mean FIC (circles) and the range of FIC (horizontal lines) are displayed. P values represent differences in the distribution of FIC as was calculated by pairwise Kolmogorov-Smirnov test. [file 1755-8794-3-47-S5.DOC]

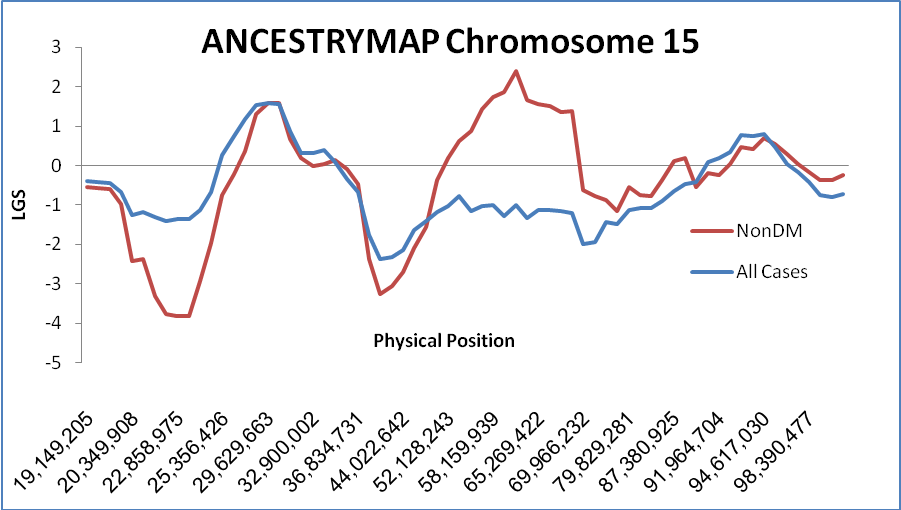


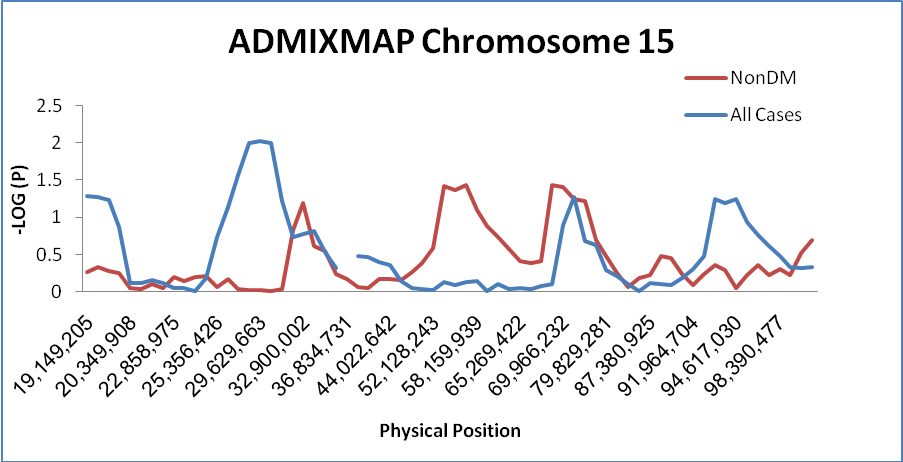

Supplement: Additional file 6 — Chromosome 15 LGS and - LOG (P) scores of the screening panel by ANCESTRYMAP and ADMIXMAP respectively. LGS and - LOG (P) are provided by ANCESTRYMAP and ADMIXMAP respectivly for AIMs along chromosome 15. Using the screening panel a total of 576 ESKD AA patients (Red line) were genotyped of whom 299 ESKD patients did not have diabetes mellitus (NonDM) (Blue line). [file 1755-8794-3-47-S6.DOC]
