# Supplementary material for: Admixture mapping of end stage kidney disease genetic susceptibility using estimated mutual information ancestry informative markers
Source: BMC Med Genomics. 2010 Oct 18;3:47. doi: 10.1186/1755-8794-3-47 (PMC2975638; doi:10.1186/1755-8794-3-47)
Supplement: Supplementary file 4 [file 1755-8794-3-47-S3.DOC]

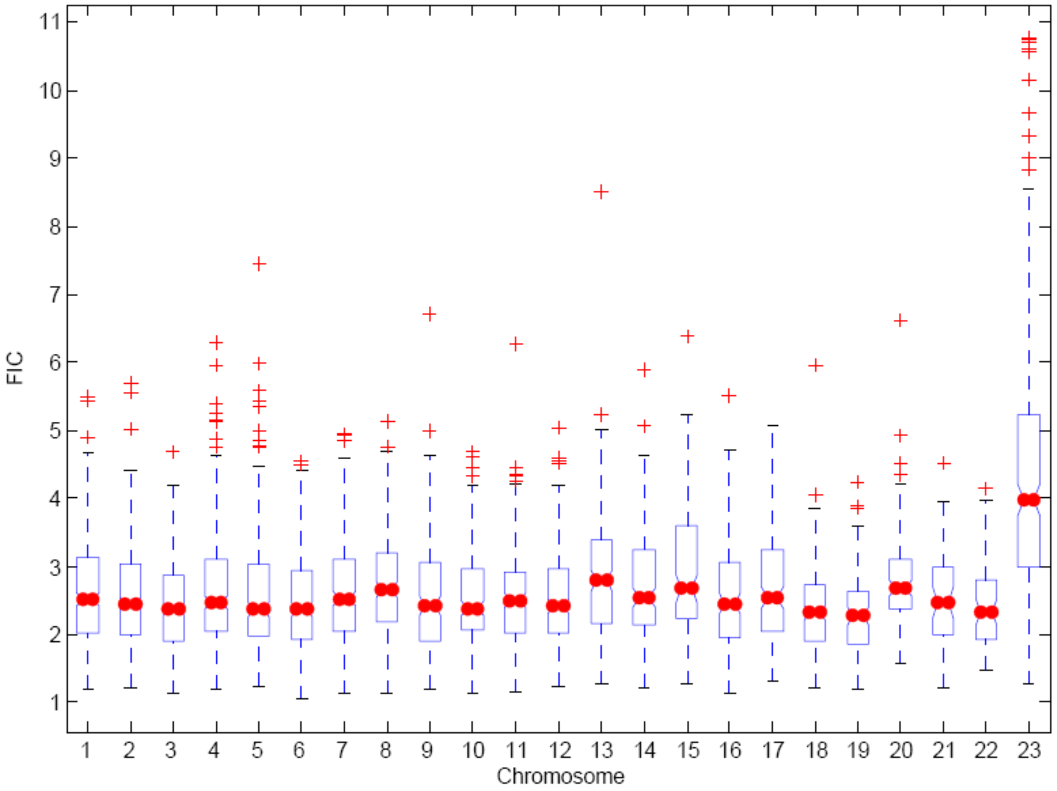
FIC
Chromosome
1 2 3 4 5 6 7 8 9 10 11 12 13 14 15 16 17 18 19 20 21 22 23
1 2 3 4 5 6 7 8 9 10 11
