# Supplementary material for: Admixture mapping of end stage kidney disease genetic susceptibility using estimated mutual information ancestry informative markers
Source: BMC Med Genomics. 2010 Oct 18;3:47. doi: 10.1186/1755-8794-3-47 (PMC2975638; doi:10.1186/1755-8794-3-47)
Supplement: Supplementary file 5 [file 1755-8794-3-47-S4.DOC]

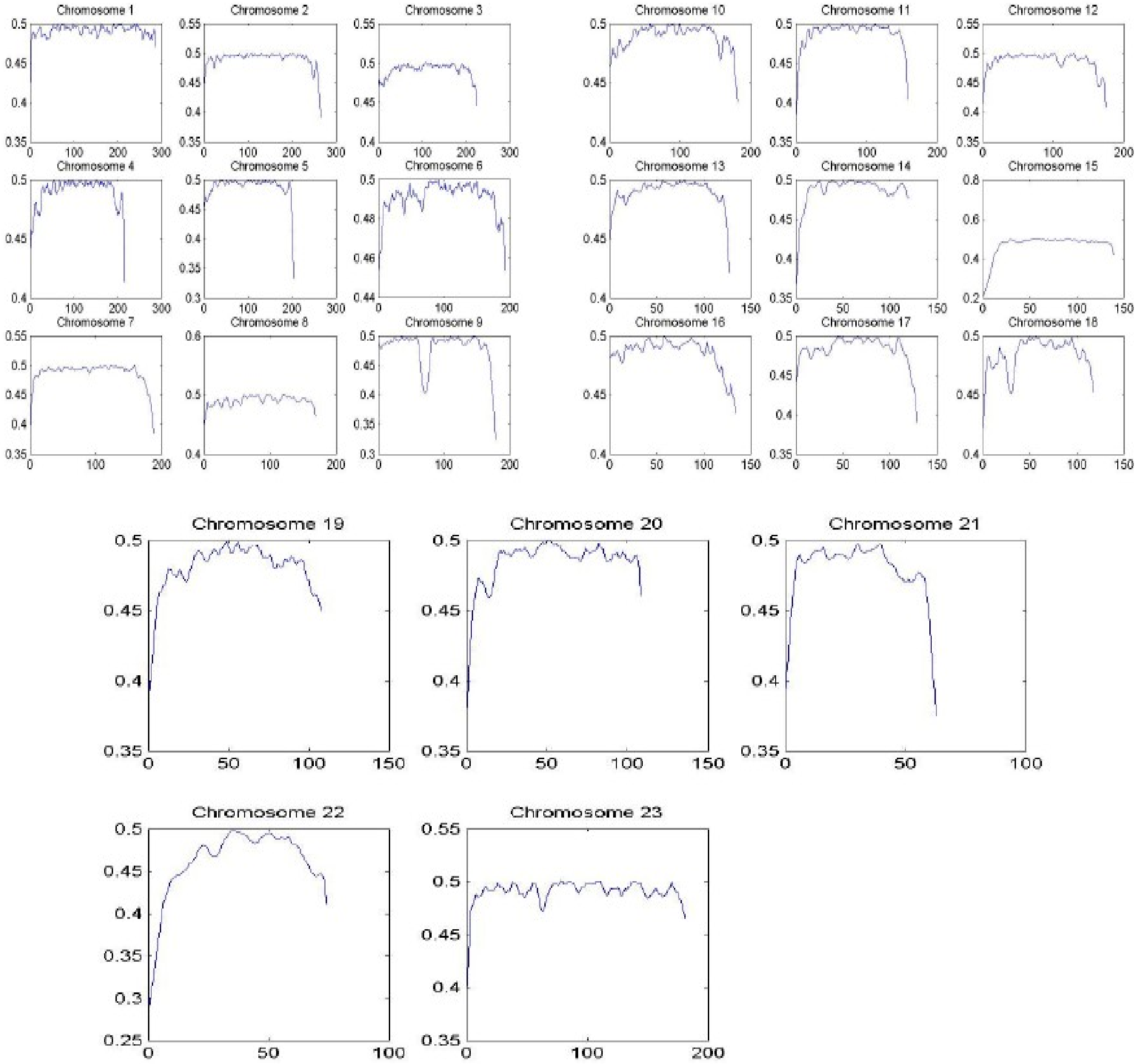
Chromosome 1
Chromosome 2
Chromosome 3
Chromosome 4
Chromosome 5
Chromosome 6
Chromosome 7
Chromosome 8
Chromosome 9
Chromosome 10
Chromosome 11
Chromosome 12
Chromosome 13
Chromosome 14
Chromosome 15
Chromosome 16
Chromosome 17
Chromosome 18
Chromosome 19
Chromosome 20
Chromosome 21
Chromosome 22
Chromosome 23
